# Enhancing service availability and resource deployment in IoT using a shared service replication method

**DOI:** 10.1016/j.heliyon.2024.e25255

**Published:** 2024-02-01

**Authors:** Khaled Kaaniche, Salwa Othmen, Ayman Alfahid, Amr Yousef, Mohammed Albekairi, Osama I. El-Hamrawy

**Affiliations:** a Department of Electrical Engineering, College of Engineering, Jouf University, Sakakah, 72388, Saudi Arabia; b Department of Computers and Information Technologies, College of Sciences and Arts Turaif, Northern Border University, Arar, Saudi Arabia; c Department of Information Systems, College of Computer and Information Sciences, Majmaah University, Majmaah, 11952, Saudi Arabia; d Electrical Engineering Department, University of Business and Technology, Ar Rawdah, Jeddah, 23435, Saudi Arabia; e Engineering Mathematics Department, Alexandria University, Lotfy El-Sied st. off Gamal Abd El-Naser, Alexandria, 11432, Egypt; f Electrical Engineering Department, Faculty of Engineering, Suez Canal University, Ismailia, 41522, Egypt

**Keywords:** Data availability, IoT, NFV, Regression learning, Resource utilization, Service availability

## Abstract

In many real-world contexts, the Internet of Things (IoT) is valued for its capacity to facilitate the smooth operation of interoperable applications and services. It is critical to ensure the accessibility and replication of IoT resources to improve the agility of these applications. As a solution, the Network Function Virtualization (NFV) paradigm is embedded into the IoT design to leverage information from various endpoint applications better and maximize resource utilization. In this study, the Shared Replication Augmenting Method (SRAM) is proposed to increase resource usage in underutilized NFVs and maintain service availability simultaneously. The regressive decision-making learning used by SRAM enables the detection of NFV needs for data and application portability across various real-time use cases. This regression method can uncover data needs and their causes, allowing for prompt answers and more efficient use of available resources. The suggested SRAM technique dynamically modifies the procedure while considering computation-less function allocations, making it suitable for various interoperable applications. It distributes root-to-service virtualization and availability based on historical use and data replication. Therefore, SRAM improves resource usage by 7.09 % with no increase in latency or delays. It also increases service availability by 10.4 %, reduces latency by 11.89 %, eliminates backlogs by 11.1 %, and reduces data repetition by 8.97 %. This study enhances resource consumption and productivity in IoT settings by offering SRAM as a viable solution. The study's results prove its potential to reduce the occurrence of replication, delay, and queues while raising the availability of services.

## 1 Introduction

The Internet of Things is a fast-growing technology widely used in many smart applications to enhance the performance of their users and help them provide better services [1]. The IoT-based application offers a better experience and helps to increase the number of users [2]. Wireless sensor networks (WSN) are used in IoT to compute the sensors used to identify the exact positions of the users. Every network system has the complex and time-consuming challenge of identifying the available IoT services [3]. A base station (BS) is where every service is stored and processed before being given to the users. A priority-aware security availability scheme is used in the IoT to ensure the security and privacy of the users while providing services [4,5]. The plan uses an unmanned aerial network to determine the users' whereabouts, which helps to make the services more accessible to customers. The unmanned area vehicle (UAV) system assignment algorithm is used in the service availability scheme to determine the needs and provide services at the required time to the users [6,7]. Every location is assigned based on the service assignment algorithm and produces the finest dataset for the server to form an accurate priority based on the users' needs. The Cloud computing system is used in the IoT service availability process by optimizing the actual characters of the users' requests. The application then provides services to the user [8].

IoT connects a larger number of people with minimum requirements and in less time. IoT provides many services to give a better experience to users. IoT-based applications to users widely provide services. However, not all services are valued or appreciated by users [9]. The service replication model is used in IoT applications to overcome this issue. The replication model identifies the storage space of the database and finds out the remaining space to perform the service. The service replication models increase the overall quality of service (QoS) and network performance [10]. The fog computing-based service replication model is widely used in the IoT to enhance the stability and scalability of the network. It uses the FogSim stimulator to identify the location and cause of the request given by the users [11,12]. It helps to find out the exact location and need for the request and helps to secure the storage space in the database. It reduces the overall latency rate while providing service, which helps increase the network's performance [13]. A service replication scheme using a proactive sensing mechanism is used in the IoT to improve service availability and increase the response time for providing services to users. It helps to increase the efficiency and performance of IoT networks [14,15].

IoT is widely used in smart homes, smart devices, and applications to better users' services by understanding their actual needs. Virtualization plays a crucial part in enhancing network efficiency by virtualizing all of the network's hardware. Real-time scenarios require a variety of interoperable apps, and virtualization is essential to boosting network efficiency in these situations [16].

The SRAM study's motivation was to find a way to improve service availability in IoT applications beyond what was previously possible with the available technologies. Investigating SRAM as a novel strategy to enhance the efficiency of IoT services was driven by the clear identification of inefficiencies and the necessity for proactive, user-centric, and flexible methods. An innovative strategy for enhancing service availability in Internet of Things (IoT) applications is the Shared Replication with Allocation Management (SRAM) mechanism. It incorporates virtualization demand and time-dependent allocation into its decision-learning-based resource allocation optimization, which involves proactively recognizing procedures that accumulate data. SRAM optimizes latency, prevents data replication, guarantees high service availability, and efficiently controls backlogs. It can be easily integrated into different IoT contexts due to its adaptability. The SRAM is an innovative approach to enhancing service availability in Internet of Things (IoT) applications. Its features include the following: proactive prevention of replication, allocation of resources with the user in mind, dynamic adaptation to user density, optimization of latency and allocation dependent on time, management of backlogs, and integration of decision-learning. With SRAM, data replication is decreased, and resource utilization is increased. Because of its adaptability, it may be used in many tech environments, guaranteeing robust and efficient service improvement in IoT applications.

The logical abstraction of hardware available on the network is the virtualization process. Two main schemes are used to solve virtualization and produce better communication for the users. Network Function Virtualization (NFV) and software-defined network (SDN) models. NFV and software-defined networks have improved resource utilization under computation-less function allocations [17]. NFV is a process that abstracts the functions from hardware to a virtual environment, such as data balancing and calculations. It provides a better way to replace the dedicated hardware with commodity servers, which helps to generate better service to the users [18]. NFV is interrelated with SDN, which helps boost the network and control the data flow of the services. SDN is used as a decision-making process for the device to understand the exact hardware features available in the network. SDN controls the network's traffic flow and controls the data flow while processing data from one server to another. An SDN modifies the network's operations and routing configuration to create a programmable network. SDN raises the network's performance, functionality, and efficiency, which raises the bar for the network [19,20].

Intelligent traffic light control and congestion prediction are two decision-making applications in a smart city that rely on real-time traffic data. SRAM is useful because it can pinpoint critical pieces of information and selectively duplicate them across the network. As a result, there is less waiting time, and more data is available for decisions to be made in real-time. Additionally, SRAM optimizes resource allocation and virtualization, which prevents data processing backlogs. It also optimizes service availability by mirroring vital traffic data and adjusting to user density and available resources. This method lessens latency, increases service availability, and improves the availability of real-time traffic statistics. The main contributions to this paper are:

• To develop SRAM for interoperable applications and shared services across different real-time IoT environments,
• To perform a mathematical computation based on decision-making learning to improve the resource allocation ratio and service availability.
• To reduce high latency and backlogs and improve resource utilization through precise data allocation,
• The method's effectiveness relies on resource utilization, less data replication, less latency, fewer backlogs, and higher service availability.

The remainder of the essay is structured as follows: The research on the virtualization strategy in the IoT architecture for various application terminals is discussed in Section 2 by many researchers. Section 3 explains the Shared Replication Augmenting Method (SRAM) for non-overloaded NFVs in concurrent service availability. Section 4 evaluates the method's effectiveness using resource utilization, less data replication, less latency, fewer backlogs, and higher service availability. The conclusion has been discussed in Section 5 and the future research scope.

## 2 Related works

Ren et al. [21] proposed an orchestration scheme for Internet of Things (IoT) service function chains (IoTSFC). The proposed method uses a multi-criterion-based algorithm for computing. The suggested solution uses a mixed-integer linear programming paradigm to identify any issues that may arise throughout the data processing process. SFC aids in improving the system's overall efficiency and protecting users from intruders. Roy et al. [22] introduced a context-aware fog-enabled scheme for Internet of Things (IoT) based applications. Fog nodes are used here to reduce the network's computation time and system delay. An optimization problem is solved, and the computation process's latency rate is minimized using a machine learning algorithm. The proposed strategy improves the system's overall performance, according to experimental findings. A dynamic service function chain embedding strategy for NFV-enabled IoT applications using deep reinforcement learning (DRL) was proposed by Fu et al. [23]. SFC is a combination of NFV and virtual network functions (VNF), which helps to reduce the number of problems which are occurred in IoT networks. DRL algorithm is used here to handle complex problems embedded in IoT applications.

Nkenyereye et al. [24] introduced a virtual framework for the IoT slice function using an edge computing system. The suggested architecture aids in developing appropriate network-slicing technology to offer users a better network. This program uses an efficient elastic computing algorithm to improve its services and performance, contributing to its overall scalability and efficiency. Niu et al. [25] proposed a workload allocation mechanism for IoT applications using an edge computing system. This method helps to reduce the system delay rate and provides safe and secure services to the users. A swarm algorithm is used to identify the problems using edge nodes. The edge computing process does the workload allocation process. The suggested framework improves the workload allocation process, according to experimental findings, and lowers the system delay rate. Xu et al. [26] presented a novel ARVMEC scheme, which stands for adaptive recommendations of a virtual machine for IoT in an edge-cloud environment. The prediction process is done using a tree-based ensemble learning algorithm. The suggested ARVMEC enhances the application's overall efficiency and boosts prediction accuracy compared to existing frameworks. ARVMEC provides users with the best VM recommendation, allowing their budget or deadline constraints. Selecting the suitable VM configuration for the respective workload on resource utilization can efficiently increase performance and reduce costs which ARVMEC needs to design appropriately.

Karatas et al. proposed a fog-based data distribution service (F-DAD) for IoT applications [27]. The system's efficacy and efficiency are improved by using F-DAD to locate the optimized issue and offer a better resolution to the application. According to experimental findings, the proposed F-DAD system has superior performance and minimizes computing latency. The huge amount of IoT data processing with less service availability has been considered a significant challenge that needs efficient and effective optimization. Further, the proposed model helps optimize the efficient and effective placement of data generated using distributed IoT nodes. Abbasi et al. [28] proposed a workload allocation scheme for IoT-based applications using an edge computing system. The allocation procedure is processed using a genetic algorithm (GA), which can manage a bigger volume of data. GA is used to improve the performance and general quality of service of IoT-based apps. While processing data, the suggested way reduces latency. According to experimental findings, the proposed strategy improves the allocation process's effectiveness, benefiting all services' quality.

Haque et al. [29] invented a new resource-aware SDN-based IoT application. Optimizing the NFV nodes makes it easier to deliver suitable services to users and decreases system lag. The proposed approach improves overall performance over other current systems by lowering system computation costs and energy consumption rates. A fuzzy interface-based approach for multi-integer linear programming (MILP) in IoT networks was put out by Farooq et al. [30]. It helps find out the process and provides the needed cache process to the network. The migration process is used to learn more about the network's specifics, which aids in giving users better performance. Users are given enhanced service using micro-cache, which improves the network's overall reliability. A novel IoT edge-cloud federation (IoTEF) for a fault-tolerance system was introduced by Javed et al. [31]. IoTEF pinpoints edge node failure tolerance and offers a more effective fix. IoT architecture is essential for locating the problem nodes and lowering the latency rate for data processing. The proposed IoTEF approach, according to experimental findings, improves performance and quality while lowering latency and ensuring connectivity between the hardware and software.

A software-defined network (SDN)-based optimal service (OSO) method was put forth by Hao et al. [32] for use in Internet of Things applications. Here, the edge nodes are optimized using a non-dominated sorting genetic method, which improves overall performance by speeding up computation. The suggested solution improves the general efficacy and flexibility of the network for consumers in comparison to previous offloading schemes. Bali et al. [33] introduced a lightweight service scheme for efficient edge nodes in IoT applications. The clustering strategy is used to identify edge nodes, which increases the amount of services that are offered to users. Edge nodes are the main cause for improving the overall performance and scalability of the device, and they provide better communication service to the users. The proposed method improves the device's efficiency and adaptability in comparison to other existing techniques. The complexity, latency and attack resilience with more backlogs of authentication schemes remain the most difficult challenges. The findings demonstrate that the suggested lightweight service scheme outperforms previous techniques in terms of attack resistance, communication cost, and time cost.

Abbasi et al. [34] proposed a workload allocation scheme for the IoT using a multi-objective genetic algorithm for fog-cloud architecture. The proposed method helps to reduce the latency rate and system delay rate. A genetic algorithm is used here for the workload allocation process, which helps find out the problems that occur in fog nodes and provides a better solution to the network. According to experimental findings, the suggested strategy improves system latency and energy consumption rate, boosting network performance.

Zahra and Chishti [35] intended to critically evaluate contemporary approaches to IoT security and pinpoint key elements of any successful security posture in the field. They suggest GLSF2 IoT, a generic and lightweight security mechanism that uses fuzzy logic and fog to detect malicious behavior in ambiguous IoT contexts. According to the results, the GLSF2 IoT mechanism offers an effective and reliable security solution for IoT systems. It provides scalability, minimal resource consumption, and improved accuracy in identifying malicious behavior while addressing important security concerns like uncertainty and insider threats. The proposed approach holds great promise for enhancing IoT system security posture and ensuring the long-term advancement of IoT technology.

Dogea et al. [36] outlines integrating a sensor capability into an IoT architecture and installing it in aircraft wings to increase component dependability. The methodology uses five layers of IoT architecture to provide effective data collecting, pre-processing, and visualization for real-time monitoring and analysis, each fulfilling a distinct function. A wireless sensor network is carefully positioned in the wings, and the data gathered is pre-processed to weed out noise and guarantee accuracy. The information is integrated into three IoT platforms: ThingSpeak on the cloud and MATLAB® on a desktop computer. Monitoring aircraft wing performance and environmental factors in real-time during flight is helpful. Key results are improved dependability, smart ecosystem support, proactive maintenance, and in-flight services.

Umair et al. [37] intended to create a human comfort-focused, energy-efficient home automation system. It uses a Markov chain probabilistic model to collect user activity patterns, which is then used to forecast energy use. The proactive energy conservation algorithm (PF-PEC) is proposed to reduce energy consumption while maintaining typical human comfort. A genuine smart home is used to validate the fog-based IoT architecture strategy. The results demonstrate the effectiveness of the PF-PEC algorithm and Markov-chain-based probabilistic model for maximizing energy economy in home automation systems while emphasizing user comfort. The significant energy savings gained show its potential for real-world use and contribute to user-centered, sustainable smart home environments.

Picone et al. [38] concentrated on utilizing Digital Twins (DTs)-based IoT systems to offer data-driven services and support improved control and decision-making. It investigates the possibility of distributed methods in which cloud and edge computing work well together. As a fresh tactical component for developing distributed cyber-physical applications, they present the idea of edge digital twins (EDT). The results show that the Edge Digital Twins (EDT) paradigm is a viable strategy for IoT systems. EDT provides efficient interaction of IoT devices and services by expanding the role of Digital Twins and applying them on the edge. This promotes interoperability, enhances last-mile digitalization, and facilitates digitalization. The experimental analysis supports the EDT model's efficacy and demonstrates its potential for use in diverse IoT applications.

Snehi et al.'s method for analyzing the utility of virtualization in resolving problems with Software-Defined Networking (SDN) and Network Function Virtualization (NFV) in cloud computing techniques for Internet of Things (IoT) infrastructures and services was put forward in their paper at [39]. By utilizing data from IoT applications and network operations, the authors hope to present an SDN-based IoT architecture that surpasses conventional network protocols' drawbacks. The results show that the suggested SDN-based IoT architecture can improve networks by utilizing virtualization, edge cloud technologies, and knowledge-driven methodologies. The study emphasizes how crucial it is to handle network issues and incorporate cloud technologies with SDN and IoT to build IoT systems that are more effective and flexible.

Metaheuristics such as the Improved Parallel Genetic Algorithm (IPGA) were put out by Wu et al. [40] and are utilized in simulation settings such as synthetic fog computing. The inclusion of elitist operators serves to avert local optima. Metrics for performance include Trust Management Mechanism and Quality of Service (QoS). The simulations make use of the synthetic fog environment. Benefits include being mindful of latency, cost, and trust. Additionally, the method deals with SPP as a problem with several objectives. However, its efficacy may be configuration- and operator-specific, necessitating validation in real-world settings.

The Bayesian Learning Automata (BLA) was suggested by Farahbakhsh et al. [41] to improve the offloading method by acquiring network states and activities. Aware of Context As part of the offloading process, the relevant application, request, sensor, resource, and network tool contexts are considered. Energy consumption, execution cost, network utilization, delay, and fairness are some of the performance parameters measured in a simulated environment, which is used to evaluate the proposed context-aware offloading algorithm. The simulation environment attempts to reproduce real-life situations using simulated context data. Using BLA learning to optimize the offloading method is one of the many benefits of this approach, along with better efficiency and performance metrics. It may be necessary to conduct real-world validation of the study due to its limitations, which include reliance on simulations and algorithm sensitivity.

Mohamed et al. [42] tackled the issue of dynamic data replication in fog computing settings by utilizing the Aquila Optimizer (AO) Algorithm and the Elephant Herding Optimization (EHO) Algorithm. The study focuses on criteria relating to data replication but does not mention specific datasets. One of the many benefits of this innovative hybrid strategy is its ability to optimize for more than one target at once. It includes load balancing, cloud throughput, the slightest cost way, and optimal path selection. Unfortunately, the paper doesn't provide details on the dataset or thoroughly address any possible problems or limitations with the suggested approach.

The comparative studies of various researches in the literature are tabulated in Table 1. The study delves into the implementation of SRAM, a computationally intensive mechanism for decision-making learning. On the other hand, the approach has been fine-tuned for computational feasibility and efficiency. The study also seeks to integrate seamlessly with varied IoT infrastructures and address compatibility difficulties with existing systems. Considering that SRAM's efficacy may differ across various IoT applications and settings, the research has tested it in multiple contexts. The study acknowledges the need for further research to investigate its applicability across a broader range of Internet of Things applications; however, it does reveal substantial gains. This research also concentrates on determining the contextual elements that impact its performance because SRAM might work well in some settings while falling flat in others.

**Table 1:** Comparative studies of existing researches in the literature.

Author	Proposed Method	Findings	Research Gaps
Dogea et al. [36]	Sensor integration into IoT for aircraft wings	Improved dependability, smart ecosystem support, proactive maintenance, and in-flight services. Monitoring aircraft wing performance and environmental factors in real-time during flight.	Long-Term Reliability and Maintenance and User Experience and Operator Training-
Umair et al. [37]	Markov chain model, PF-PEC algorithm	Significant energy savings in a human comfort-focused, energy-efficient home automation system. Effectiveness of the PF-PEC algorithm and Markov-chain-based probabilistic model for maximizing energy economy in home automation systems.	Lack of real-world use details, potential limitations in different environments.
Picone et al. [38]	Digital Twins (DTs), Edge Digital Twins (EDT)	Edge Digital Twins (EDT) as a viable strategy for IoT systems, promoting interoperability, enhancing last-mile digitalization, and facilitating digitalization. Experimental analysis supports EDT model's efficacy in diverse IoT applications.	Lack of comparative analysis with existing approaches and scalability considerations.
Snehi et al. [39]	SDN-based IoT architecture, Virtualization	Improvement of networks by utilizing virtualization, edge cloud technologies, and knowledge-driven methodologies. Emphasis on handling network issues and incorporating cloud technologies with SDN and IoT for more effective and flexible IoT systems.	Lack of details on specific improvements, challenges in real-world implementations.
Wu et al. [40]	Improved Parallel Genetic Algorithm (IPGA)	Latency-aware approach with IPGA in synthetic fog computing. Benefits include considering latency, cost, and trust. Dealing with Service Placement Problem (SPP) as a multi-objective issue.	Efficacy may be configuration- and operator-specific, requires validation in real-world settings.
Farahbakhsh et al. [41]	Bayesian Learning Automata (BLA)	Context-aware offloading algorithm using BLA learning, addressing energy consumption, execution cost, network utilization, delay, and fairness in a simulated environment. Benefits include efficiency and improved performance metrics.	Need for real-world validation due to simulation reliance and algorithm sensitivity.
Mohamed et al. [42]	Aquila Optimizer (AO), Elephant Herding Opt.	Dynamic data replication using AO and EHO algorithms in fog computing. Optimization for multiple targets, including load balancing, cloud throughput, the slightest cost way, and optimal path selection.	Lack of dataset details, insufficient addressing of potential problems or limitations with the proposed approach.

## 3 Proposed shared replication augmenting method (SRAM)

In [26], ARVMEC provides users with the best VM recommendation, which ARVMEC needs to design appropriately for effective resource utilization. Further, in Ref. [27], the huge amount of IoT data processing with less service availability has been a significant challenge that needs efficient and effective optimization. The complexity, latency, and attack resilience with more backlogs of authentication schemes remain the most difficult challenges in Ref. [32]. Therefore, based on the firm analysis, the SRAM has been designed and developed for detecting NFV-required interoperable applications to optimize resource utilization, less data replication, less latency, fewer backlogs, and higher service availability.

**Fig. 1:**
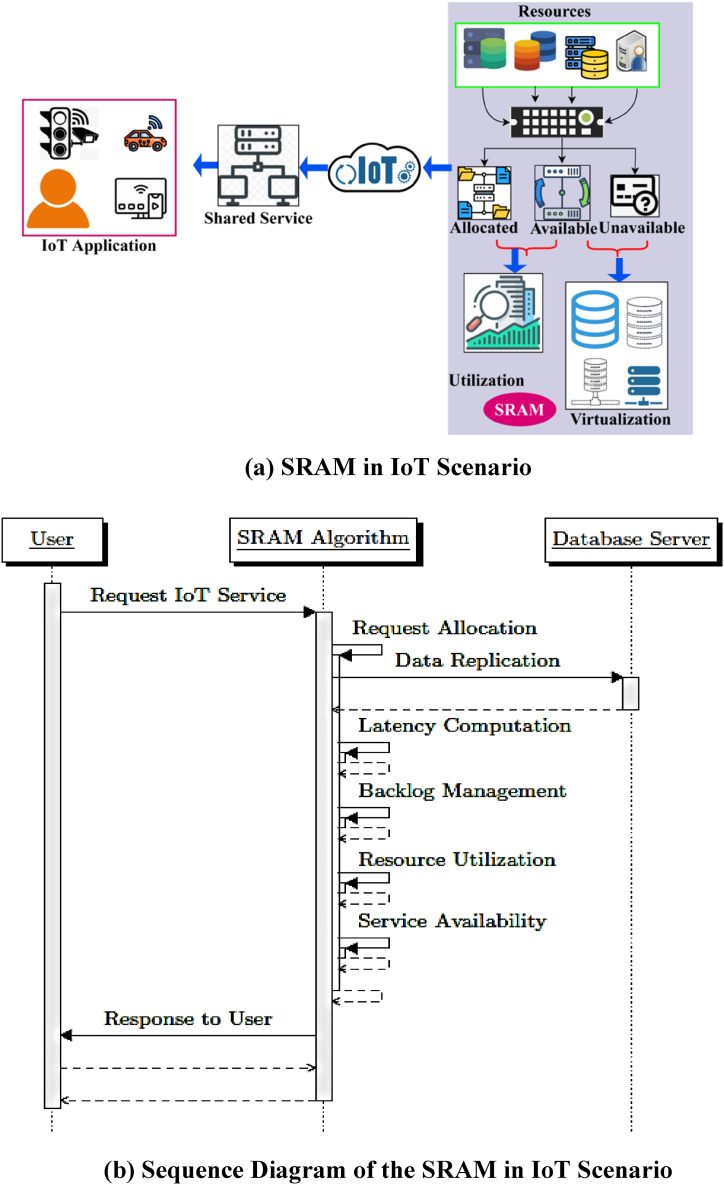
(a)SRAM in IoT ccenario. (b) Sequence diagram of the SRAM in IoT scenario.

The main goal of this model of SRAM is to leverage the interoperable applications and shared services of different real-time IoT environments, as shown in Fig. 1.

In this IoT architecture, the utilization and virtualization of the different service applications are examined to improve the resource utilization of shared services. Different shared service applications of users from smart cities, business, manufacturing, transportation, mobility, automotive, medicine, healthcare, etc., require replication services. Therefore, data availability of the service and the total number of users, trustability in shared services, and replication utilization are important considerations (refer to Fig. 1(a) and (b)). The proposed SRAM mainly focuses on this type of replication by providing entire application terminals for replication utilization through NFV-required resource management. In this proposed framework, data availability is improved for users and their duties with the existing service providers. IoT users access their service resources through utilization and virtualization using their mobile applications. The SRAM model is designed to function between the service providers and IoT applications. In this framework, allocation of available and unavailable resources is done through computing to easily achieve an augmentable solution for the different duties and services. This model's primary objective is providing shared service responses without delay and maximizing available resources. In particular, replicating this data from IoT storage must be implemented efficiently for the objectives of high availability and disaster recovery. To that aim, we examine the challenge of uploading IoT data from a series of sensor gateways and effective replication of this data on distributed shared service storage in this paper. While there are other attempts in this domain, they either do not examine replication in the context of small size and many data items inherent in IoT data, or they focus on access time after replication based on availability and unavailability. SRAM is predicated on the availability of many distributed data centers, mini-Clouds, from which data may be copied for utilization. This proposed framework functions in two segments: resource utilization and virtualization (replication) concurrent and service availability.

(1) $$u{d_{r}}_{m}=L_{R_{p}}-L_{R_{q}}$$

In Eq. (1), let assumes, $\alpha_{i}$ is represented as the availability of $n^{th}$ resources of tasks at t, service request $R_{p}$ and service response is $R_{q}$. For every request $R_{q}$, response $R_{p}$ is allocated by analyzing from the maximum availability of the resources $\alpha_{n}$. The resource allocation is denoted as $\max_{n\in t}\alpha_{n}\;\forall \;R_{p}=R_{q}$. During the response allocation process $R_{p}$, the responding time ($u{d_{r}}_{m})$ is computed for every request ($R_{q})$. The response access time ($ud_{r}L_{R_{q}}$) should be minimum ($\min_{m\in R_{q}}u{d_{r}}_{m}\;\forall \;R_{p})$ while accessing the request $({ud_{r}L}_{R_{p}}$).

The objective of diminishing the replication is performing with the variable $\beta_{n}\;\forall \;n\in R_{p}$. If $U_{N}=\left\{1,2,\ldots ,U_{N}\right\}$ denotes the classes of users and then the total quantity of tasks in the accessing time $\left(xd_{t}\right)$ is $R_{p}\times t$. Where the access request is $U_{N}\times R_{p}$. As per the overall service request of $U_{N}\times R_{p}$, $t\times R_{p}$ are the attainable tasks for achieving. Data allocation from the service level operations and users is functioning and spreading for analyzing the resource center. The application terminals communicate with n={1,2,…N} classes of users; these users are capable of providing data from all the operation levels of the smart industry applications. The above-mentioned n communicates with a different data allocation in different time interval $t=\left\{1,2,\ldots d_{t}\right\}$ and the replicated application response $\left(A_{R}\right)$ is estimated as

(2) $$A_{R}=\left\{ \begin{array}{c} n\times R_{p}\times d_{t}\;\forall \;n∷t\;\text{and}\;B=0\\ u_{n}\times \frac{N-B}{n}\times d_{t}\forall \;\left(n,B\right)∷t\;\text{and}\;B\neq 0 \end{array}\right.$$

**Fig. 2:**
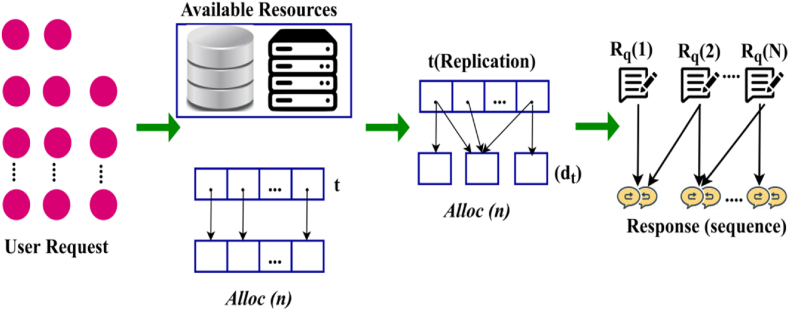
Application response sequence.

In Eq. (2), Let B represent the number of backlogs terminal pursued in the smart industry applications. Based on the process, the total number of resources per unit time be $R_{p}u_{n}$ and $u_{d}$ denotes the backlogs and high latency in t, n∷t and (n,B)∷t specifies the swiftness of application response $R_{p}$ and user backlog terminals to the different time intervals t. The n∷t is estimated as $\sum_{i=1}^{n}{R_{p}}_{i}$ and the (n,B)∷t is computed as $\sum_{i=1}^{N}{R_{p}}_{i}-u_{n}\sum_{i=1}^{B}{R_{p}}_{i}\text{.}$ During the computation, backlog values are estimated from the ${R_{p}}_{u_{d}}$, latency value $u_{d}$ and request related response $R_{p}$; $u_{n}\text{.}$ The data replication from the resources is hidden in the application terminal and IoT architecture for leveraging the data availability. In the different application terminals, consequences of data and $A_{R}$ are make sure of gathering information for the swift response t is succeeded. For gathering resources, the non-mining terminal requires applications and data requirements. The NFV application of information between N∈n and B is operated using the accessibility of their IoT applications and swift timing. In Eq. (3), the condition B>N produces less inadequate data from the shared service resources.

(3) $$\begin{array}{c} {d_{t}}_{B}=\sum_{i=1}^{N}\frac{\alpha_{n}}{{d_{t}}_{n}}\\ \text{such}\;\text{that}\\ \Delta A_{R}=\frac{A_{R}}{\left(N-B\right)}-\left(\alpha_{n}-u_{d}\right) \end{array}\}$$

where the variables ${d_{t}}_{B}$ and $\Delta A_{R}$ denote the swift response and routine of gathering data. The swift responses for the shared service and the sequence $A_{R}$ depends on $\left(N\times R_{p}\right)$ are the resource sharing conditions for replication. The feasible replication of the resource sharing instance $\left(R_{s}\right)$ is determined for each sequence of t; this estimation is observed for accessing the condition B≠0 and B=0 for all different time intervals t using recursive decision-making learning. Fig. 2 presents the application response sequence for replicated instances.

The user requests are provided with the available resources based on Alloc(n), wherein the replication process is required. The replication is performed for t and $d_{t}$ (Refer to Fig. 2) for the response to be sequential. Contrarily, if the available requests are non-sequential, then $R_{q}$ for 1 to N under different decisions. The recursive analysis is based on a sequence of gathering data $R\;{d_{t}}_{B}$ and $\Delta A_{R}$ such that $R_{p}$ is defined in all the intermediate data requirements.

The user request by sending results to Alloc(n). The available resource displays the results to the replication nodes and tracks activities based on t and $d_{t}$. The information about these activities is provided who can store it in an observation database and utilize it in future encounters to generate more qualitative suggestions based on response sequence.

The linear output of $\Delta A_{R}$ in ${d_{t}}_{B}$ is the allocated resource for utilization analysis for increasing $\left(N\times R_{p}\right)$. The variable Q and final result X is important in defining $R_{q}$. The inputs from the IoT applications for the analysis of $A_{R}$ for both n∷t and (n,B)∷t that has different time intervals. In this allocation of resources, the distribution of shared services $\left(R_{p}\times t\right)$ for all n based on $\alpha_{n}$ is the detecting NFV analysis. The probability of allocating resources $\left(\rho_{t_{B}}\right)$ consecutively is derived in Eq. (4) as

(4) $$\begin{array}{c} \rho_{t_{B}}={\left(\;1-L_{r}\right)}^{i-1}\;\forall \;i\in t\\ \text{such}\;\text{that}\\ L_{r}=\left(1-\frac{R_{p}\in n}{R_{p}\in t}\right) \end{array}\}$$

The consecutive representation, as in Equation (4), implies the stable likelihood of n such that there are no outstanding resources, and hence the anticipated allocation time Et in Equation (1). Hence, the resource allocation for $\rho_{t_{B}}$ are as follows

(5) $$Alloc\;\left(n\right)=\frac{1}{\left|R_{q}-R_{p}+1\right|}\;.{\left(\rho_{t_{a}}\right)}^{i}\;\forall \;i\in t$$

In Eq. (5), $P_{t_{a}}$ is computed from the request and response allocation according to the specific allocation time and allocation operation X(θ) That is defined in Eq. (6).

(6) $$P_{t_{a}}=\frac{\rho_{t_{B}}.\;Alloc\;\left(n\right)*\left[\left(R_{q}-R_{p}\right)L_{r}-\left(\frac{R_{q}-R_{p}}{n}\right)\frac{et}{t_{r_{q}}}\right]X\left(\theta \right)}{X\left(\theta \right)*n}$$

here X(θ) is defined as $\int_{0}^{t}{Et}^{t-1}{\left(1-Et\right)}^{t-1}\;d\left(Et\right)$ which is computed for every response allocation $X\left(\theta \right)\;\forall \;Alloc\;\left(n\right)=\int_{1}^{R_{p}}{Et}^{t-1}.\frac{L_{r}}{{d_{t}}_{B}}{\left(1-\rho_{t_{B}}\right)}^{t-1}\;d\left(R_{p}\right)$.

However, the resource allocation for n as in the above equation is satisfied for both $\left(u_{n}\times R_{p}\right)$ and $\left(t\times R_{p}\right)$ make sure on-time service response. The conjunction method of access of regression process of assigning t is to be reducing the high latency of the swift response condition $\left(u_{n}\times R_{p}\right)>\left(t\times R_{p}\right)$. The resource allocation is expressive using the data requirements, and their root is identified for providing the fast response. Therefore, the identifying condition of $u_{n}>t$ and $\rho_{t_{B}}$ is decrease to valid the above equation. Here, the execution time results in less latency. Where, the variable condition X(θ) represents the allocation operation for t. From all the resource allocation processing, the regression in assigning services to the n is an overloading problem. As mentioned above, the shared resource allocation requires more resource utilization and reduces the latency in a service delay. The available resource is the representation of the regression process based on $u_{n}>t$ and n non-overloading NFVs in the concurrent service availability. The control dissemination is addressable using regressive decision-making learning is to detect the impacts of NFV required applications and data requirements through the replication process. The following segment denotes the available data utilization process to detect the following impact. The solution for replicating the available data utilization process depends upon regressive decision-making learning. It helps to detect NFV applications and data requirements in the concurrent instance. The replication process relies on different metrics for accessing the non-overloading and high latency probability at shared resource allocation. Therefore, the condition for shared resource allocation is varied, regulating the regression process through replication. The utilization is assigned for both the condition mentioned above and then computing the n available resources probability and allocation of resources for planning time. The first available data utilization relies on maximum backlogs ${d_{t}}_{B}$ and X(θ) as

(7) $$\begin{array}{c} X\left(\theta ,{d_{t}}_{B}\right)=\left[R_{q}-\left(\frac{Et}{t_{R_{p}}}\right)\times \frac{1}{n}\right]-Alloc\;\left(n\right)+1\;\\ \text{such}\;\text{that}\\ \begin{array}{c} \sum_{n\in t}\sum_{m\in R_{p}}t_{nm}-\sum_{k\in R_{q}}d_{t}\\ \text{and}\\ n_{i}=\sum_{i\in t\;}Alloc\;\left(n_{i}\right)-{\left(P_{t_{a}}\right)}_{n} \end{array} \end{array}\}$$

**Fig. 3:**
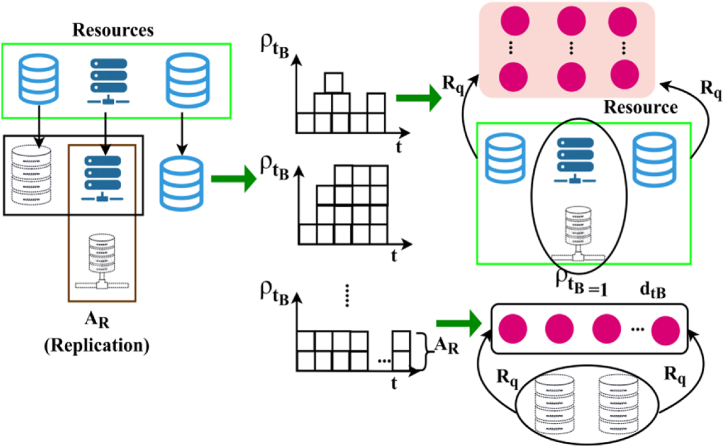
Allocation based on replicated $A_{R}$.

As per the above Equation (7), the backlogs depends upon the allocation of the resources for the above condition, as in $P_{t_{a}}$ and Alloc(n). The allocation based on replicated $A_{R}$ is illustrated in Fig. 3.

Fig. 3 illustrated that the resource allocation according to the replicated $A_{R}$. The replication of resources are examined for every user request by computing the $\rho_{t_{B}}$ concerning the task t and $d_{tB}$ for providing further allocations. This requires multiple variations and decisions on $\rho_{t_{B}}=1$ such that the unavailability conditions are discontinued (Fig. 3). Therefore, the chances of achieving unavailable resources and its sequential service are

(8) $$\begin{array}{c} P_{t_{a}}\left(t/d_{t}\right)=\frac{1}{\sqrt{2n\pi^{2}}}experssion\left[-\frac{R_{p}-L_{r}\times R_{p}}{\pi }\right]\\ and,\pi =R_{p}-L_{r}*n \end{array}\}$$

In Eq. (8), the following estimation probability, the main goal is to detect $u_{n}$ and t to reduce the resource utilization and therefore, the actual $R_{q}$ is derived as in Equation (9)

(9) $$R_{q}=\max\left\{\frac{P_{t_{a}}\times R_{p}}{Alloc\;\left(n\right)-L_{r}*R_{p}}\right\}$$

Hence, the root is the available resources $\left[1-\frac{P_{t_{a}}}{Alloc\;\left(n\right)-L_{r}*R_{p}}\right]$ and its current status in the allocation or revocation of resources. The available resource and unavailable resource jointly provide the root-to-service virtualization process, and it is based on previous utilization and data replication. The process of updating $R_{p}$ is $\left[R_{p}*X\left(\theta ,{d_{t}}_{B}\right)\right]$ is the $\beta_{i}$ previous instances, and therefore the resource utilization is reduced. The possible sequence for improving resource virtualization as per data availability is given as

(10) $$\begin{array}{c} R_{q}=\beta =R_{p}-L_{r}*n\\ maximum\;possible\;virtualization\\ \begin{array}{c} {\left(1-L_{r}\right)}^{i-1}=\frac{-R_{q}+L_{r}{.R}_{p}}{\beta^{2}}\;\forall \;i\in t\\ \begin{array}{c} R_{q}=L_{r}.R_{p}-{\left(1-L_{r}\right)}^{i-1}\beta^{2}\\ L_{r}=0,R_{q}=\beta^{2}={\left(R_{p}\right)}^{2}\;\left(\min\right)\\ L_{r}=1,R_{q}=R_{p}\left(\max\right) \end{array} \end{array}\} \end{array}$$

From the above Equation (10), the virtualization of resource utilization process at different time instances as per the response is either of β or $R_{q}\text{.}$ In this condition, if $L_{r}=0$, then $\beta =R_{p}=R_{q}$ is the maximum possible solution, and if $L_{r}=1$, then $R_{q}=R_{p}-n$/ $R_{q}=R_{p}$. Here, the root-to-service virtualization occurrence of $R_{p}=R_{q}$ is an exact solution. Therefore, the previous utilization and data replication for all the requests and responses are under computation-less function allocation, and hence the resource utilization of available resources of n is $\left(R_{p}-L_{r}*n\right)$ and ${\left(R_{p}\right)}^{1/2}$ That shows the replication and utilization time for the sequence $R_{p}$. The virtualization process determines $\left(R_{q},R_{p}\right)$ and $\left({R_{q}}_{t-1},{R_{q}}_{t}\right)$ based on accessing T from the instances. The probability of $L_{r}$ and $P_{t_{a}}$ and $\rho_{t_{B}}$ is the optimal deciding method for both types of virtualization. The replication occurrence of $\left(R_{q},R_{p}\right)$ and $\left({R_{q}}_{t-1},{R_{q}}_{t}\right)$ is differentiating based on $d_{t}$ for X(θ) is computed as

(11) $$Virtualization\;\left(n\right)=\left\{ \begin{array}{c} \frac{n-\left(L_{r}*R_{p}\right)}{n+\left(P_{t_{a}}\right)R_{p}}\;\forall \;R_{q}=R_{p}\\ \frac{n-\left(L_{r}*R_{p}\right)}{n+\left(P_{t_{a}}+\rho_{t_{B}}-L_{r}\right)R_{p}}\;\forall \;R_{q}<R_{p} \end{array}\right.$$

**Fig. 4:**
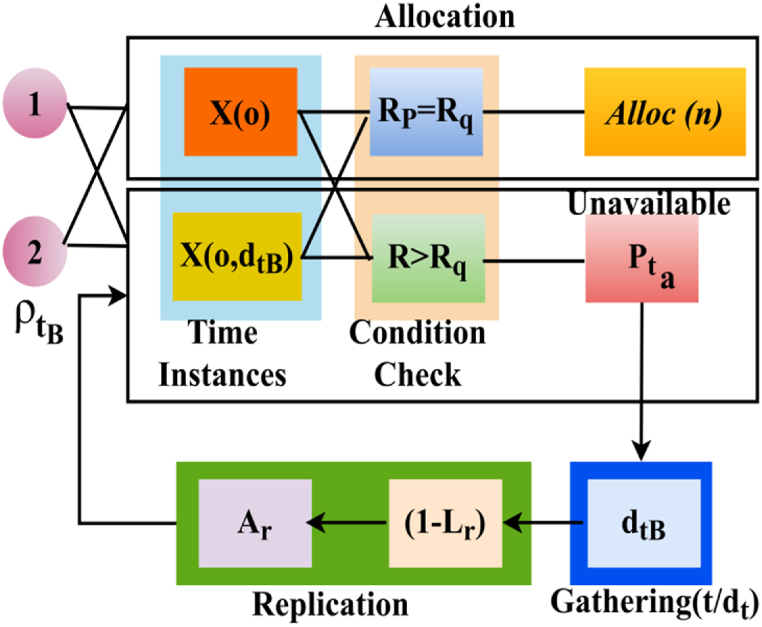
Allocation decision process.

In Equation (11), the data virtualization instance of $\left(P_{t_{a}}+\rho_{t_{B}}-L_{r}\right)$ is the stable probability of identifiable using resource Alloc(n). In Fig. 4, the allocation decision process based on learning is illustrated in Fig. 4.

The input for different sequences is given as $\rho_{t_{B}}$ from which the allocation conditions are verified. In the allocation condition, $R_{P}=R_{q}$ and $R\_>R_{q}$ are validated; the first provides precise Alloc(n), whereas the latter condition provides the resource unavailability scenario. Based on this process, the gathering is initiated for improving non-replication fewer data in $\left(1-L_{r}\right)$ providing $A_{r}$ for performing further verification. The decisions are performed based on the conditions validated in the allocation process, as presented in Fig. 4. IoT resource allocation and scheduling are critical in such a system because resource allocation and scheduling deal with the mapping between resources and nodes, as well as optimally assigning resources to available nodes. A node in an IoT data network may have to connect to hundreds of resources based on availability and unavailability. As a result, resource allocation and scheduling are processed based on time instances and conditional checks. Therefore, the actual n of available and unavailable shared resource data of responses for virtualization, the unavailable resources process until the next Et. This process is estimated as

(12) $$\begin{array}{cc} \begin{array}{c} \frac{n}{n+R_{p}}=\frac{1}{\left(R_{q}-R_{p}+1\right)}\\ n+R_{p}=nR_{q}-nR_{p}+n\\ R_{q}=\frac{\left(n+1\right)R_{-p}}{n} \end{array}| & \begin{array}{c} n-\frac{R_{p}}{n+R_{p}}=\frac{1}{\left(R_{q}-R_{p}+1\right)},as\;L_{r}=0,P_{t_{a}}=\rho_{t_{B}}=1\\ nR_{q}-R_{p.}R_{q}=R_{p}+{R_{p}}^{1}+{R_{p}}^{2}+nR_{p}\\ R_{q}=\frac{2R_{p}+n{R_{p}}^{1}+{R_{p}}^{2}}{\left(n-R_{p}\right)} \end{array}\} \end{array}$$

The unavailable resources $R_{q}\;\forall \;t\in R_{p}$ is as estimated using the above-derived Equation (12) and therefore, the following next Et is important for allocating the unavailable resources $R_{p}$. In this case of virtualization, n is the previous resource utilization and data replication irrespective of the users and services. The computation-less function allocation of IoT applications process follows either of the $R_{q}$ as in the equation mentioned above. As per the estimation in the previous section, allocation of resources for $\beta_{i}\in R_{q}=\frac{\left(n+1\right)R_{p}}{n}$ is the retaining process. The virtualization time $\left(t_{virt}\right)$ of a t in this resource allocation is the utilization metrics and its different data requirements for each n depending upon the density of processing $\left({∁}_{N}\right)$. This time is estimated as

(13) $$t_{virt}=\left\{ \begin{array}{c} \frac{{∁}_{N}}{Alloc\left(n\right)}\;\forall \;R_{q}=R_{p}/R_{q}=\frac{\left(n+1\right)R_{p}}{n}\\ \frac{{∁}_{N}}{Alloc\left(n\right)}+\frac{X\left(\theta ,{d_{t}}_{B}\right)\left(P_{t_{a}}+\rho_{t_{B}}-L_{r}\right)}{Alloc\left(n\right)},\forall \;\begin{array}{c} R_{q}<R_{p}\left(or\right)\\ R_{q}=\frac{2R_{p}+n{R_{p}}^{1}+{R_{p}}^{2}}{\left(n-R_{p}\right)} \end{array} \end{array}\right.$$

In this above Equation (13) determines, $t_{virt}\in \left[t_{R_{p}},t_{R_{q}}\right]$ and the final of $t_{virt}$ are the maximum resource allocation factor and high latency (reduce) for handling $\left(n-R_{p}\right)$ responses. Hence, the allocation of resources of all $t\in R_{p}$ improves both utilization and replication function. The initial process of virtualization of resources based on replication maximizes resource utilization and virtualization through precise data allocation. These tasks are to reduce high latency and backlogs and improve resource utilization.

### 3.1 Self-analysis

**Fig. 5:**
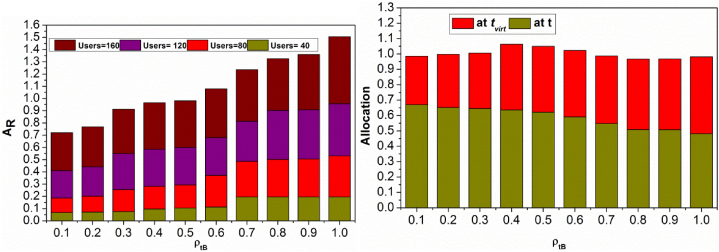
$A_{R}$ and allocation for $\rho_{tB}$.

This section presents the self-analysis for some observations by varying different factors discussed above. First, the analysis for $A_{R}$ and allocation for different $\rho_{tB}$ is presented in Fig. 5.

**Fig. 6:**
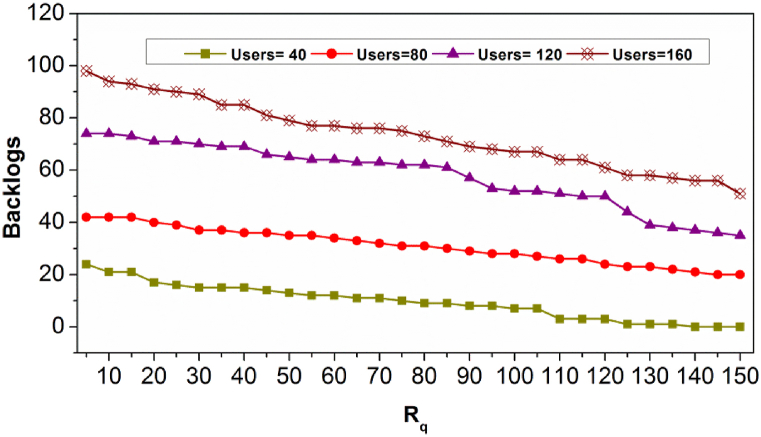
Backlogs for $R_{q}$.

In Fig. 5, the self-analysis for $A_{R}$ and allocation (at different t and $t_{virt}$) against $\rho_{t_{B}}$ is presented. The replicated resource allocation varies with the users for meeting $d_{t_{B}}$ based on Alloc(n). This improves the available resources to satisfy the demands preventing backlogs. Therefore, $P_{t_{a}}\;at\;t$ and $t_{+virt}$ is different by validating $\rho_{t_{B}}$. Depending on the user density, $R_{q}$ and virtualization (n), the allocations are planned and deployed. The $\frac{n}{n+R_{q}}$ and $\frac{R_{P}}{n+R_{P}}$ instances are alone avoided for the allocation. This requires $t_{virt}$ for allocating the above-expelled resources. Therefore, further allocations are planned across $\Delta A_{R}$ in satisfying $U_{d_{rm}}$ Thus, the allocation is differentiated under normal time and virtualization time instances for the different users. However at some $\rho_{t_{B}}=1$, the replication reaches its maximum allocation (for 40 users in Fig. 5). Fig. 6 presents the backlogs for dissimilar $R_{q}$.

**Fig. 7:**
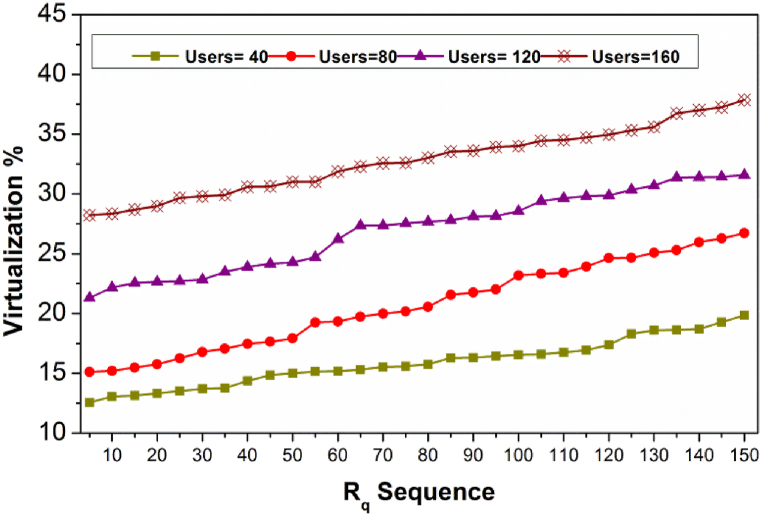
Virtualization % for $R_{q}$ sequence.

Fig. 6 illustrates the backlogs for $R_{q}$ under different users. The proposed method achieves fewer backlogs provided $R_{q}$ increases. As $R_{q}$ increases, the need for replication and $\rho_{t_{B}}$ verification is less and hence X(θ) and $X\left(\theta ,d_{t_{B}}\right)$ are congruent for reducing backlogs. The $R_{q}$ is interrupted if $P_{t_{a}}\left(\frac{t}{d_{t}}\right)$ is experienced, the case occurs if the user density increases. Fig. 7 presents the virtualization % for different $R_{q}$ sequence.

**Fig. 8:**
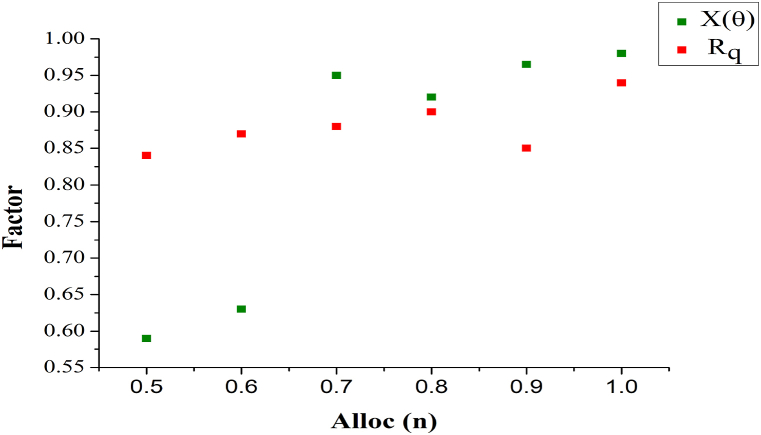
X(θ) and $R_{q}$ for Alloc(n).

Fig. 7 presents the virtualization ratio for varying $R_{q}$ and users. The virtualization ratio is maximum for controlled user density (40 and 80). Contrarily for other user densities, it increases as $\Delta A_{R}$ is high and hence if $P_{t_{a}}\left(\frac{t}{d_{t}}\right)=1$, then virtualization is high. Therefore, the applicable $d_{t_{B}}$ is achieved by X(θ) in the first allocation pursued by ${\;R}_{q}$ admission. In the consecutive $X\left(\theta ,d_{t_{B}}\right)$, the virtualization is required for meeting the user requests and thereby $P_{t_{a}}$ less. In Fig. 8, the X(θ) and $R_{q}$ for different Alloc(n).

The X(θ) and $R_{q}$ for different Alloc(n) is presented in Fig. 8. The $A_{R}$ is expected in different instances for improving the $R_{P}=R_{q}$ a requirement in t and $t_{virt}$. This is required for distributing services without facing $P_{t_{a}}\left(\frac{t}{d_{t}}\right)$. Therefore the X(θ) is always high without considering the $R_{q}$. The interrupting $R_{q}$ is required for deviating the change in multiple X(θ) factors, reducing backlogs. Hence the allocation att and $t_{virt}$ balances the above without pausing the user demand. This maximizes the X(θ) for which tallying $R_{q}$ is required.

## 4 Results and discussion

The metrics of data replication, latency, backlogs, resource consumption, and service availability are used in this section to describe the performance of the suggested solution. The Contiki Cooja simulator validates the suggested method [44]. In the open-source scenario, 240 users are accounted for, of which 14 resources are exploited. The resources are replicated in a 1:4 ratio to respond to 1000–1400 requests. The virtualization requires time, which is modeled between 90 and 210 s. The above metrics are compared with the F-DAD, ARVMEC, and RBAS methods discussed in the related works section.

### 4.1 Data replication

**Fig. 9:**
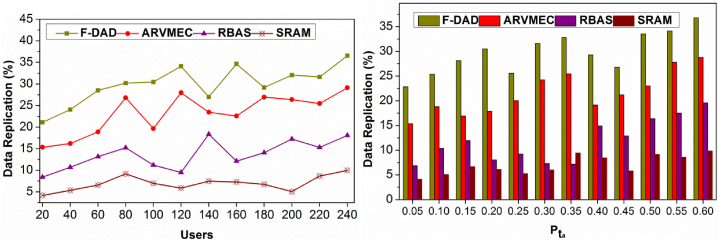
Data replication (%) comparisons.

The comparative analysis for data replication for various users and $P_{t_{\alpha }}$ is depicted in Fig. 9. In the suggested method, $A_{R}$ in t and $R_{P_{i}}\forall \;i\in N$ is estimated under multiple instances for identifying replications.

The proposed method monitors the occurrence under different identifications under the condition B>N such that $\Delta A_{R}$ sequences (causing replication) are computed. This estimation is performed until B≠0 is achieved. If $\left(u_{n}\times R_{P}\right)>\left(t\times R_{P}\right)$ is achieved, then the deviating factors determine the Alloc(n). Hence the Alloc(n) is performed for preventing data replications. In this non-replication case, $P_{t_{a}}\left(.\right)$ is verified for preventing further data replications. Therefore as the user density increases, data replications are confined. Contrarily, if $P_{t_{\alpha }}$ increases the replication due to virtualization (n) increases. This is to be confined based on the learning recommendations for preventing additional replications. The $Alloc\;\left(n_{i}\right)-{\left(P_{t_{a}}\right)}_{n}$ is validated in assigning resources to the requesting users for meeting Equation (1) requirements. The proposed method identifiers the chances based on $\left(\frac{t}{dt}\right)\;\forall \;i\in N$ and hence the replications are confined. Thus for the unavailable probabilities, the proposed method confines replications based on $\Delta A_{R}$.

### 4.2 Latency

**Fig. 10:**
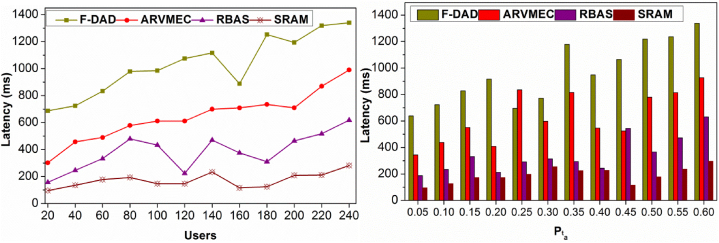
Latency (ms) comparisons.

Fig. 10 presents the latency observed in resource allocation under different users and $P_{t_{a}}$. The proposed method provides services based on replications and resources availability. In contrast to the existing method, resource availability is ensured high for treating backlogs. In different scenarios, the requirement in Equation (1) is achieved by delivering $d_{t_{B}}$.

The proposed method first identifiers the need for $A_{R}$ and hence $d_{t_{B}}$ is utilized under distinct $U_{n}$ and t. At this juncture, if the $\frac{n}{n+R_{q}}$ occurs, then $\left[R_{P}*X[\theta ,d_{p_{B}})\right]$ is relied on for replicating the resources. Therefore, the time for access and allocation is restricted within the permitted interval. This is accounted in different $\beta_{i}\;\forall i\in N$ and hence the maximum possible $\beta^{2}={\left(R_{P}\right)}^{2}$ under minimum and maximum validation increases the availability for preventing service allocation latency. Thus for different users $P_{t_{a}}$, the latency is comparatively less.

### 4.3 Backlogs

**Fig. 11:**
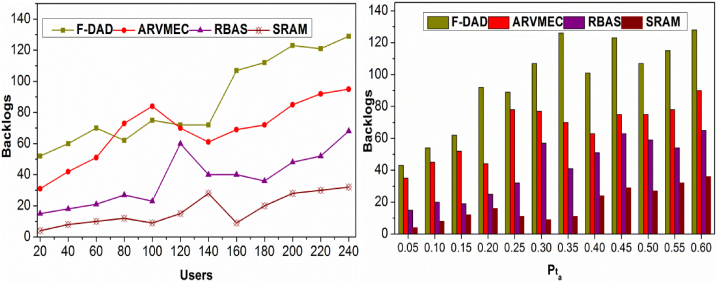
Backlogs comparison.

Fig. 11 presents the comparative analysis for backlogs analyzed for varying users and $P_{t_{a}}$. The proposed method identifies $A_{R}$ and $\rho_{t_{a}}\left(\frac{t}{d_{t}}\right)$ is verified in multiple instances for $X\left(\theta ,d_{t_{B}}\right)$ validation, preventing backlogs. The decision-making learning instances improve the virtualization (n) for $R_{q}$ and hence $L_{r}$ is reduced. In the backlog identification process, the previous decisions identify un-available resources. This detection is distinct for $R_{q}=R_{P}$ and $R_{q}<R_{P}$ and hence the Alloc(n) is modified for further allocations. The proposed method is struck in $\left(P_{t_{a}}+\rho_{t_{B}}-L_{r}\right)$ instance due to $d_{t_{B}}$ and hence the ${\left(1-L_{r}\right)}^{i-1}$ is deviated from distinct instances. The separated instances are detected and allocated based on virtualization (n) instances. Therefore, the further validations until next Et is performed based on $R_{q}$ and its allocation from previous $R_{P}^{1}$ and $R_{P}^{2}$ occurrences. The current allocations are performed depending on the $\beta_{i}\in R_{q}$ and hence the availability is maximized. This is pursued in the occurring instances in varying different instances, and hence, the backlogs are comparatively less.

### 4.4 Resource utilization

**Fig. 12:**
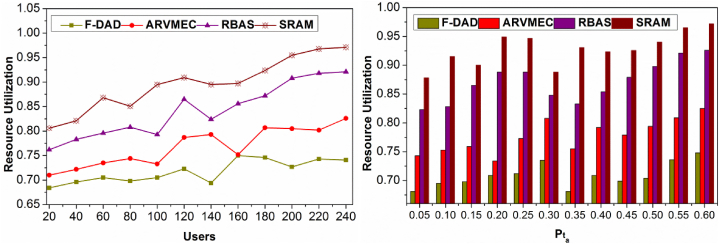
Resource utilization comparisons.

Fig. 12 shows a comparison of resource use for various users and $P_{t_{a}}$. The allocation is performed for satisfying the conditions in Equation (1). The B is mitigated depending on $R_{p_{i}}\;\forall \;i\in N$ and $u_{n}$ and hence the allocation is confined for B>N instances. In the intermediate processing $\Delta A_{R}$, the proposed method achieves fair $\rho_{t_{B}}$ allocation depending on distinct X(θ).

However, the X(θ)∀n is modified for $u_{n}>t$ condition wherein $X\left(\theta ,d_{t_{B}}\right)$ is reassigned in $d_{t}$. This increases the resource utilization in different instances, improving $R_{q}$. Contrarily, the detected $U_{n}$ reduces the resource utilization without deviating the $R_{q}$. Therefore, maxAlloc(n) in $\beta_{i}$ is achieved under high data availability such that $R_{q}=\beta$ and the same condition β is validated for $L_{r}$. The condition verification is achieved for multiple instances improving resource utilization. Besides, the virtualization (n) is required for differentiating the actual $R_{q}$, for reducing the $R_{q}<R_{P}$ occurrence. Therefore, the virtualization instances provide multiple delegations ensuring high data availability. This is common for different users and $P_{t_{a}}$ such that the unavailability is less and hence the resource availability is high.

### 4.5 Service availability

**Fig. 13:**
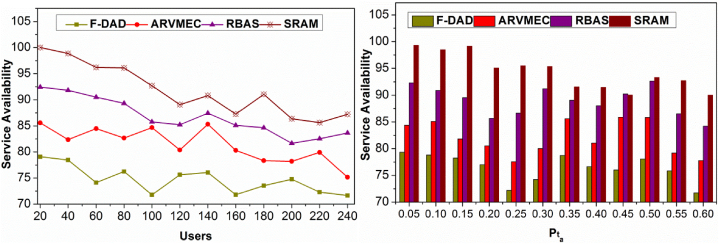
Service availability comparisons.

The proposed method achieves high service availability for different users and $P_{t_{a}}$. In this method, X(θ) is performed in t for mitigating B=0 condition and hence the $\Delta A_{R}$ is maximized. The $R_{P_{i}}$ is performed under distinct intervals for maximizing multiple $u_{n}$; this requires (n,B)∷t. The requirement is performed under distinct intervals for improving $\frac{a_{n}}{d_{t_{n}}}$. This feature reduces the B condition and improves $L_{r}$; However, the $R_{q}$ under the $\left(L_{r}-1\right)$ is required for preventing $P_{t_{a}}\left(\frac{t}{d_{t}}\right)$ in t. The above condition is prevented by providing $X\left(\theta ,d_{t_{B}}\right)$ and hence the $A_{R}$ is pursued distinct instances. Therefore in $\left(N\times R_{P}\right)$, the available resources are alone replicated, and hence the availability is retained for satisfying Equation (1) in time t. In the other contrary, the $\alpha_{n}$ instances are identified for improving the allocation rate. This is recurrent, if $P_{t_{a}}$ is observed, and hence the available Alloc(n) is modified under different intervals. In this method, the available $X\left(\theta ,d_{t_{s}}\right)$ is predicted under multiple instances for improving the availability. This process is repeated post $P_{t_{a}}$ Identification and B≠0 conditions. Therefore, the proposed method achieves high resources available for different users and $P_{t_{a}}$ as presented in Fig. 13. Hence SRAM helps to improve the resource utilization of 7.09 %, without high latency and backlogs. In addition, it achieves 8.97 % less data replication, 11.89 % less latency, 11.19 % fewer backlogs, and 10.4 % higher service availability.

This research compares the SRAM method to other methods and assesses its efficacy in data replication, latency, backlogs, resource consumption, and service availability. Although future studies will include statistical tests to demonstrate the statistical relevance of these improvements, results reveal a reduction in replication percentages. The statistical significance of the discrepancies will be confirmed through hypothesis testing. Additional evaluations will use statistical analyses to determine the importance of these differences, although the method demonstrates better resource consumption efficiency. The study highlights the significance of using statistical tests to conduct a more thorough review. An essential part of deploying IoT services is the effect of the SRAM method on resource costs. This assessment looks at how it performs in various situations, considering user densities, request intensities, and resource demands. The objective is to determine the impact on the total resource cost of the SRAM's adaptation to changes in these parameters. Its resource cost implications are contrasted with well-established methods such as F-DAD, ARVMEC, and RBAS to assess if the approach offers a more economical alternative. Ensuring the evaluation's robustness is done by statistical validation, which uses hypothesis testing to verify the statistical significance of observed discrepancies. By considering resource costs across many scenarios and validating results through statistical studies, the following review intends to comprehend the SRAM method's economic efficiency thoroughly.

## 5 Conclusion

This article introduced a method for augmenting shared replication to improve service availability in various IoT application requirements. This method identifies the data-accumulating routines and resources allocation probability based on user density in the forehand. Based on the recommendations of decision learning, the proposed method performs data utilization and prevents resource unavailability. The available probability is matched using the virtualization demand and the allocation at different time intervals. In this method, decision-making learning is employed to improve the resource allocation ratio and service availability. Regardless of the user densities and resource unavailability, the decision-making process instigates the time-dependent allocation, preventing data replication from the accumulated sequence. Therefore, the latency in allocation is confined, whereas the availability probability is maximized. The proposed method achieves 8.97 % fewer data replication, 11.89 % less latency, 11.19 % fewer backlogs, 7.09 % higher resource utilization, and 10.4 % higher service availability for the different user densities.

## CRediT authorship contribution statement

**Khaled Kaaniche:** Data curation, Project administration, Writing – original draft, Writing – review & editing. **Salwa Othmen:** Resources, Writing – review & editing, Conceptualization. **Ayman Alfahid:** Formal analysis, Funding acquisition, Writing – review & editing. **Amr Yousef:** Validation, Visualization, Writing – review & editing. **Mohammed Albekairi:** Investigation, Methodology, Writing – review & editing. **Osama I. El-Hamrawy:** Data curation, Visualization, Writing – review & editing.

## Declaration of competing interest

The authors declare that they have no known competing financial interests or personal relationships that could have appeared to influence the work reported in this paper.
